# Supplementary material for: Ocular delivery of lipid nanoparticles-formulated mRNA encoding lanosterol synthase ameliorates cataract in rats
Source: Nat Commun. 2025 Sep 26;16:8522. doi: 10.1038/s41467-025-63553-5 (PMC12474983; doi:10.1038/s41467-025-63553-5)
Supplement: Supplementary file 1 — Supplementary Information [file 41467_2025_63553_MOESM1_ESM.pdf]

Ocular delivery of lipid nanoparticles-formulated mRNA encoding lanosterol synthase ameliorates cataract in rats

Ruiteng Song,[1] Yongqi Lin,[2] Min Zhang,[3] Zhen Liu,[1] Rui Zhang,[1] Jun Zhao,[3] and Bin Li[1,2,*]

[1]Department of Infectious Disease, Shenzhen People's Hospital, The Second Clinical Medical College, Jinan University, Shenzhen 518020, China

[2]School of Medicine, Southern University of Science and Technology, Shenzhen, 518055, China

[3]Department of Ophthalmology, Shenzhen People's Hospital, The Second Clinical Medical College, Jinan University, Shenzhen, 518020, China

[*]Corresponding author: libin@mail.sustech.edu.cn

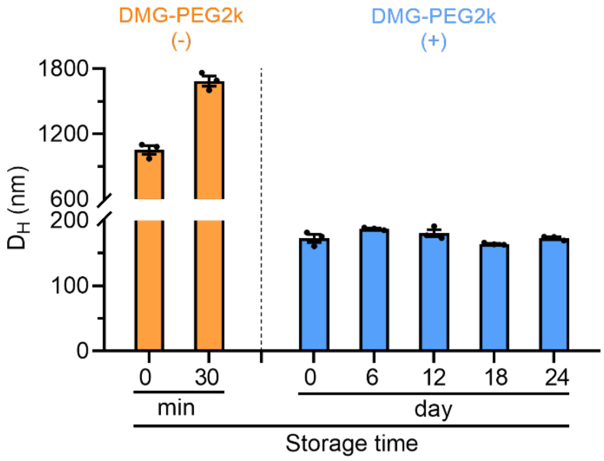

Supplementary Fig. 1 Effects of DMG-PEG2k on the particle size of mRNA formulations over time. Data are presented as mean ± SEM (n = 3 technical replicates). Source data are provided as a Source Data file.

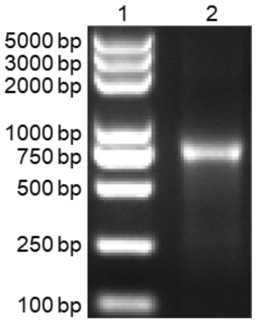

Supplementary Fig. 2 Purity of 5moU-modified hLSS mRNA determined by 1% native agarose gel. Lane 1, DNA ladder. Lane 2, 5moU-modified hLSS mRNA.

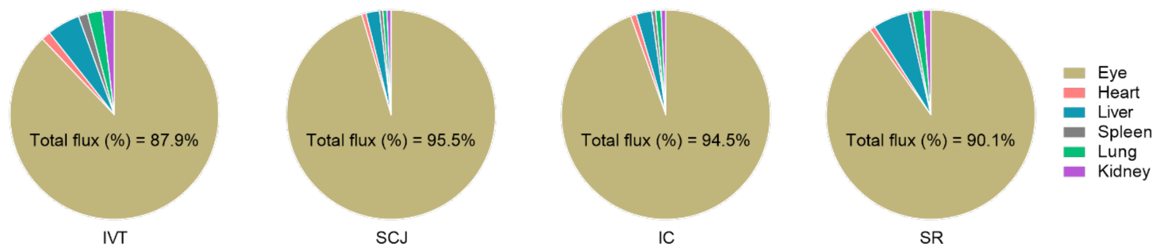

Supplementary Fig. 3 Percentage of luminescence flux in the eyes based on the ex vivo tissues (eye, heart, liver, spleen, lung, and kidney) examined 4 h after injection of FLuc mRNA-loaded pB-UC18 LNPs.

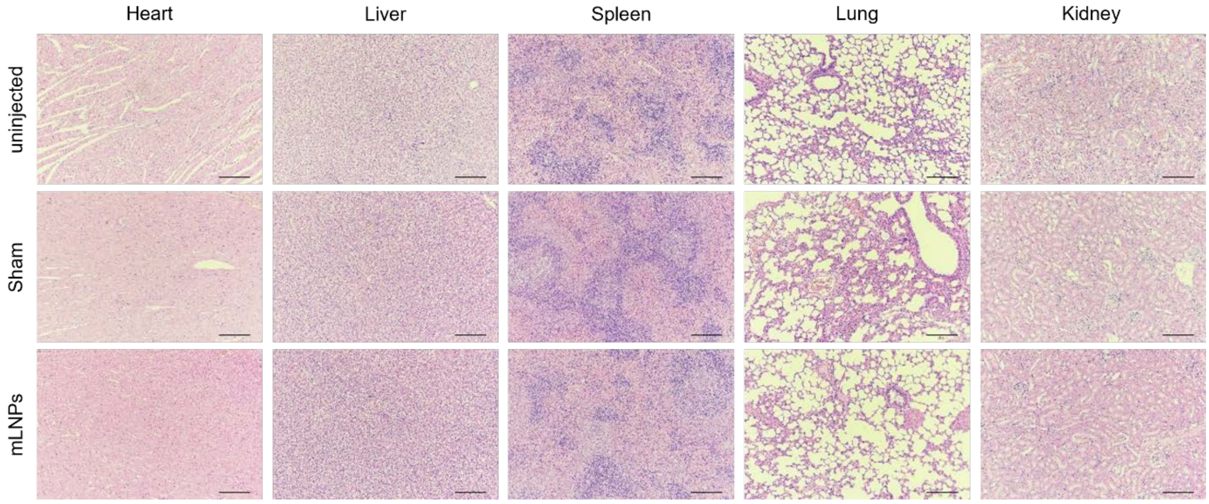

Supplementary Fig. 4 Images of H&E-stained tissue sections (heart, liver, spleen, lung, and kidney) from the uninjected, sham, and mLNPs groups. Scale bar, 200 μm.

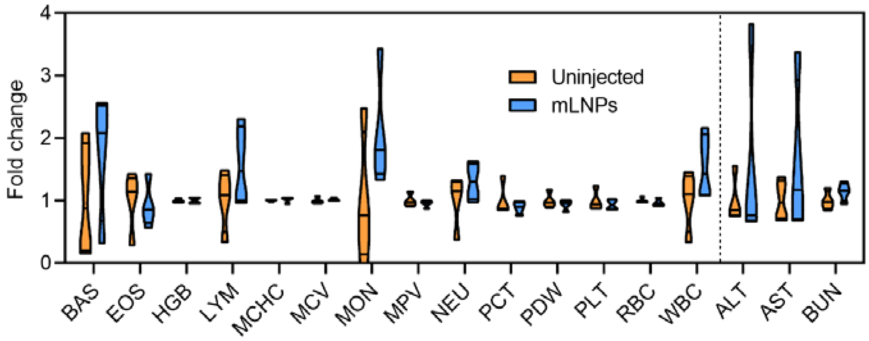

Supplementary Fig. 5 Routine blood (the first 14 parameters) and hepatic and renal function (the last 3 parameters) test results from the uninjected and mLNPs groups. Data are presented as mean ± SEM (*n* = 4 rats, two-way ANOVA with Sidak's multiple comparison test). Violin plots indicate minimum, lower quartile, median (middle line), upper quartile, and maximum. No statistically significant difference was observed for all parameters tested. BAS, basophil. EOS, eosinophil. HGB, hemoglobin. LYM, lymphocytes. MCHC, mean corpuscular hemoglobin concentration. MCV, mean corpuscular volume. MON, monocyte. MPV, mean platelet volume. NEU, neutrophil. PCT, plateletcrit. PDW, platelet distribution width. PLT, platelets. RBC, red blood cell. WBC, white blood cell. ALT, alanine aminotransferase. AST, aspartate aminotransferase. BUN, blood urea nitrogen. Source data are provided as a Source Data file.

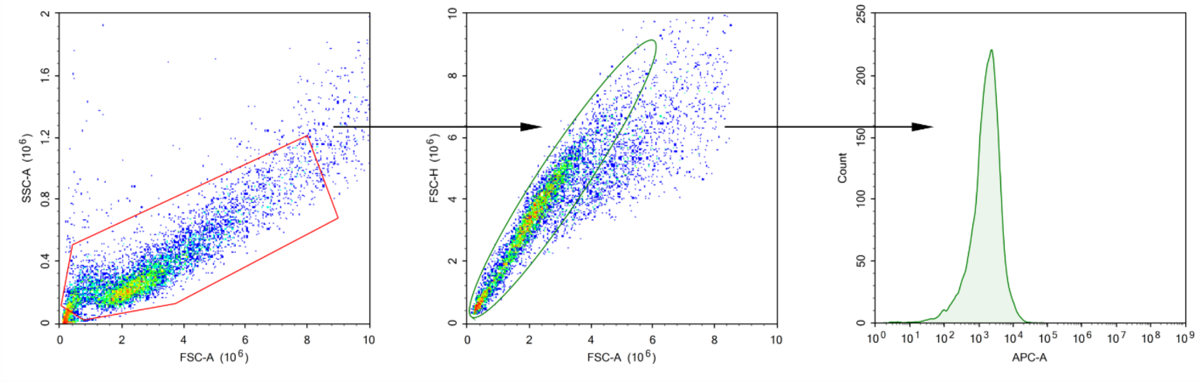

Supplementary Fig. 6 Gating strategy for analysis of the mean fluorescence intensity (MFI) in Fig. 2e.

Supplementary Table 1 Protein sequence of hLSS.

MTEGTCLRRRGGPYKTEPATDLGRWRLNCERGRQTWTYLQDERAGREQTGLEAYALGLDTK
NYFKDLPKAHTAFEGALNGMTFYVGLQAEDGHWTGDYGGPLFLLPGLLITCHVARIPLPAG
YREEIVRYLRSVQLPDGGWGLHIEDKSTVFGTALNYVSLRILGVGPDDPDLVRARNILHKK
GGAVAIPSWGKFWLAVLNVYSWEGLNTLFPEMWLFPDWAPAHPSTLWCHCRQVYLPMSYCY
AVRLSAAEDPLVQSLRQELYVEDFASIDWLAQRNNVAPDELYTPHSWLLRVVYALLNLYEH
HHSAHLRQRAVQKLYEHIVADDRFTKSISIGPISKTINMLVRWYVDGPASTAFQEHVSRIP
DYLWMGLDGMKMQGTNGSQIWDTAFAIQALLEAGGHHRPEFSSCLQKAHEFLRLSQVPDNP
PDYQKYYRQMRKGGFSFSTLDCGWIVSDCTAEALKAVLLLQEKCPHVTEHIPRERLCDAVA
VLLNMRNPDGGFATYETKRGGHLLELLNPSEVFGDIMIDYTYVECTSAVMQALKYFHKRFP
EHRAAEIRETLTQGLEFCRRQQRADGSWEGSWGVCFTYGTWFGLEAFACMGQTYRDGTACA
EVSRACDFLLSRQMADGGWGEDFESCEERRYLQSAQSQIHNTCWAMMGLMAVRHPDIEAQE
RGVRCLLEKQLPNGDWPQENIAGVFNKSCAISYTSYRNIFPIWALGRFSQLYPERALAGHP

Supplementary Table 2 Coding sequence of hLSS mRNA.

AUGACCGAGGGCACCUGUCUGCGGCGGCGUGGAGGGCCCUACAAGACCGAGCCUGCAACCG<br>ACCUGGGCAGAUGGCGGCUGAACUGCGAGCGAGGCAGACAGACCUGGACAUAUCUGCAGGA<br>CGAGCGGGCUGGCCGGGAACAGACAGGCCUGGAAGCCUAUGCCCUGGGCCUGGAUACAAAG<br>AACUACUUCAAGGACCUGCCCAAGGCCCACACCGCCUUUGAGGGCGCUCUGAAUGGCAUGA<br>CAUUCUACGUGGGCCUACAGGCUGAGGACGGCCACUGGACCGGCGACUACGGCGGCCCUCU<br>GUUCCUGCUGCCAGGCCUGCUGAUCACCUGUCACGUUGCUAGAAUCCCCCUGCCAGCUGGU<br>UAUCGGGAAGAGAUCGUGAGAUACCUGCGCAGCGUGCAGCUGCCUGACGGAGGCUGGGGCC<br>UGCACAUCGAGGACAAGAGCACCGUGUUUGGAACCGCCCUGAAUUACGUCAGCCUGCGCAU<br>UCUGGGCGUUGGCCCCGAUGAUCCAGACCUGGUGCGGGCCAGAAACAUCCUGCACAAAAAG<br>GGCGGAGCCGUGGCUAUCCCUAGCUGGGGAAAAUUCUGGCUUGCCGUGCUCAACGUGUACA<br>GCUGGGAAGGCCUCAAUACCCUGUUCCCUGAGAUGUGGCUGUUUCCUGAUUGGGCACCUGC<br>CCACCCUUCCACCCUGUGGUGCCACUGCAGACAAGUGUACCUGCCUAUGAGCUACUGCUAC<br>GCCGUCAGACUGAGCGCCGCCGAGGACCCCCUGGUCCAGUCCCUGAGACAGGAGCUUUACG<br>UCGAAGACUUCGCCUCUAUCGACUGGCUGGCCCAGCGGAACAACGUGGCCCCUGAUGAGCU<br>GUAUACACCUCAUAGCUGGUUGCUGAGAGUGGUGUACGCCCUGCUGAAUCUGUACGAGCAU<br>CACCACAGCGCCCACCUGAGGCAAAGAGCCGUGCAGAAGCUGUACGAGCACAUCGUGGCCG<br>ACGACAGAUUCACCAAGUCCAUCAGCAUCGGACCCAUCAGCAAGACAAUCAACAUGCUGGU<br>GCGGUGGUACGUGGACGGCCCUGCCAGCACUGCCUUUCAGGAGCACGUGUCUAGAAUCCCU<br>GACUACCUGUGGAUGGGCCUGGACGGCAUGAAGAUGCAGGGAACAAACGGCAGCCAAAUCU<br>GGGACACCGCUUUUGCCAUCCAGGCCCUGCUUGAGGCCGGCGGACACCACAGACCUGAAUU<br>CUCCAGCUGUCUGCAAAAGGCCCACGAGUUCCUGAGAUUGUCCCAGGUGCCAGACAACCCU<br>CCUGACUACCAGAAAUACUACAGACAGAUGCGGAAGGGCGGAUUCAGCUUCAGCACACUGG<br>ACUGCGGCUGGAUCGUGUCUGAUUGCACAGCUGAAGCUCUGAAAGCCGUGCUGCUGCUGCA<br>AGAGAAGUGCCCUCACGUGACCGAGCAUAUCCCCAGAGAGAGACUGUGCGACGCCGUGGCC<br>GUGCUGUUAAACAUGAGAAACCCCGAUGGAGGCUUCGCCACCUACGAAACCAAAAGAGGCG<br>GCCACCUGCUGGAACUGCUGAACCCCAGCGAGGUGUUCGGCGAUAUUAUGAUCGACUACAC<br>CUACGUGGAAUGCACCAGCGCCGUGAUGCAGGCCCUGAAGUACUUCCACAAGCGGUUUCCC<br>GAACACCGGGCUGCUGAAAUCAGAGAGACACUGACCCAAGGCCUGGAAUUCUGCAGAAGAC<br>AGCAGAGAGCUGACGGCUCCUGGGAGGGCUCUUGGGGCGUGUGCUUCACCUACGGCACAUG<br>GUUCGGACUGGAAGCCUUCGCCUGCAUGGGCCAGACCUACAGAGAUGGCACCGCCUGUGCC<br>GAGGUGAGCAGAGCUUGUGAUUUCCUGCUGUCUAGACAGAUGGCCGACGGCGGAUGGGGCG<br>AGGAUUUCGAGAGCUGUGAAGAAAGAAGAUACCUCCAGAGCGCCCAGUCUCAGAUCCACAA<br>CACCUGCUGGGCCAUGAUGGGCCUGAUGGCCGUGAGACACCCCGAUAUCGAGGCCCAGGAG<br>AGAGGCGUGCGGUGCCUGCUGGAAAAGCAGCUGCCUAACGGCGAUUGGCCUCAGGAAAACA<br>UCGCCGGCGUGUUCAACAAGAGCUGCGCCAUUUCUUAUACAAGCUACCGGAACAUCUUCCC<br>AAUCUGGGCCCUGGGCAGGUUCAGCCAGCUGUACCCUGAGCGGGCCCUGGCUGGCCAUCCU<br>UGA
