# Supplementary material for: Ocular delivery of lipid nanoparticles-formulated mRNA encoding lanosterol synthase ameliorates cataract in rats
Source: Nat Commun. 2025 Sep 26;16:8522. doi: 10.1038/s41467-025-63553-5 (PMC12474983; doi:10.1038/s41467-025-63553-5)
Supplement: Supplementary file 2 — Reporting summary [file 41467_2025_63553_MOESM2_ESM.pdf]

nature portfolio

Corresponding author(s): Bin Li

Last updated by author(s): Aug 5, 2025

Reporting Summary

Nature Portfolio wishes to improve the reproducibility of the work that we publish. This form provides structure for consistency and transparency in reporting. For further information on Nature Portfolio policies, see our Editorial Policies and the Editorial Policy Checklist.

Statistics

For all statistical analyses, confirm that the following items are present in the figure legend, table legend, main text, or Methods section.

n/a	Confirmed	
☐	☒	The exact sample size (*n*) for each experimental group/condition, given as a discrete number and unit of measurement
☐	☒	A statement on whether measurements were taken from distinct samples or whether the same sample was measured repeatedly
☐	☒	The statistical test(s) used AND whether they are one- or two-sided<br>*Only common tests should be described solely by name; describe more complex techniques in the Methods section.*
☒	☐	A description of all covariates tested
☒	☐	A description of any assumptions or corrections, such as tests of normality and adjustment for multiple comparisons
☐	☒	A full description of the statistical parameters including central tendency (e.g. means) or other basic estimates (e.g. regression coefficient) AND variation (e.g. standard deviation) or associated estimates of uncertainty (e.g. confidence intervals)
☐	☒	For null hypothesis testing, the test statistic (e.g. *F*, *t*, *r*) with confidence intervals, effect sizes, degrees of freedom and *P* value noted<br>*Give P values as exact values whenever suitable.*
☒	☐	For Bayesian analysis, information on the choice of priors and Markov chain Monte Carlo settings
☒	☐	For hierarchical and complex designs, identification of the appropriate level for tests and full reporting of outcomes
☒	☐	Estimates of effect sizes (e.g. Cohen's *d*, Pearson's *r*), indicating how they were calculated

Our web collection on statistics for biologists contains articles on many of the points above.

Software and code

Policy information about availability of computer code

Data collection	Particle size and zeta potential data were collected using the Particle Solution (3.6.0.7122) software. Flow cytometry data were collected using the NovoExpress software (1.6.1). In vitro immunofluorescence images were acquired using the ZEN software (3.1.0.00003). Confocal fluorescence images were acquired using the Leica Application Suite X software (3.5.6.21594). In vivo luminescence images were acquired using the Living Image software (4.4). Immunofluorescence images from tissue sections and TUNEL images were taken using the CaseViewer software (2.4).
Data analysis	Data analysis was performed with GraphPad Prism (version 9.5.1). Western blot and slit-lamp images were quantified with the Image J software (version 1.54g).

For manuscripts utilizing custom algorithms or software that are central to the research but not yet described in published literature, software must be made available to editors and reviewers. We strongly encourage code deposition in a community repository (e.g. GitHub). See the Nature Portfolio guidelines for submitting code & software for further information.

Data

Policy information about availability of data

All manuscripts must include a data availability statement. This statement should provide the following information, where applicable:

- Accession codes, unique identifiers, or web links for publicly available datasets
- A description of any restrictions on data availability
- For clinical datasets or third party data, please ensure that the statement adheres to our policy

Source data are available for Figs. 2b, 2e, 2g, 2h, 3c, 3d, 3f, 4b, 5d, and 6c and Supplementary Figs. 1, 2, and 5 in the associated source data file. Source data are provided with this paper.

Research involving human participants, their data, or biological material

Policy information about studies with human participants or human data. See also policy information about sex, gender (identity/presentation), and sexual orientation and race, ethnicity and racism.

Reporting on sex and gender	n/a.
Reporting on race, ethnicity, or other socially relevant groupings	n/a.
Population characteristics	n/a.
Recruitment	n/a.
Ethics oversight	n/a.

Note that full information on the approval of the study protocol must also be provided in the manuscript.

Field-specific reporting

Please select the one below that is the best fit for your research. If you are not sure, read the appropriate sections before making your selection.

☒ Life sciences ☐ Behavioural & social sciences ☐ Ecological, evolutionary & environmental sciences

For a reference copy of the document with all sections, see nature.com/documents/nr-reporting-summary-flat.pdf

Life sciences study design

All studies must disclose on these points even when the disclosure is negative.

Sample size	Sample sizes were determined according to the pilot studies, previous literatures, and practical considerations. No statistical methods were used to predetermine sample size. The details of the sample size are provided in the figure legends and where relevant.
Data exclusions	No data were excluded.
Replication	Experiments were repeated and experimental findings were reproducible.
Randomization	All samples or organisms were randomly allocated into experimental groups.
Blinding	Investigators were not blinded to group allocation during data collection and analysis as no subjective data collection or scoring was required.

Reporting for specific materials, systems and methods

We require information from authors about some types of materials, experimental systems and methods used in many studies. Here, indicate whether each material, system or method listed is relevant to your study. If you are not sure if a list item applies to your research, read the appropriate section before selecting a response.

Materials & experimental systems

n/a	Involved in the study	
☐	☒	Antibodies
☐	☒	Eukaryotic cell lines
☒	☐	Palaeontology and archaeology
☐	☒	Animals and other organisms
☒	☐	Clinical data
☒	☐	Dual use research of concern
☒	☐	Plants

Methods

n/a	Involved in the study	
☒	☐	ChIP-seq
☐	☒	Flow cytometry
☒	☐	MRI-based neuroimaging

Antibodies

Antibodies used	Antibodies used in western blot analysis:<br>Mouse monoclonal antibody against β-actin (Beyotime, Cat No. AA128).<br>HRP-conjugated goat anti-mouse IgG (Beyotime, Cat No. A0216).<br>Rabbit polyclonal antibody against LSS (Proteintech, Cat No. 18693-1-AP).<br>HRP-conjugated goat anti-rabbit IgG (Abclonal, Cat No. AS014).<br><br>Antibodies used in immunofluorescence analysis:<br>Rabbit polyclonal antibody against LSS (Proteintech, Cat No. 18693-1-AP).<br>FITC-labeled goat anti-rabbit IgG (Abclonal, Cat No. AS011).<br>Alexa Fluor 488-labeled goat anti-rabbit IgG (Servicebio, Cat No. GB25303).
Validation	Commercially available antibodies are used for the specified applications (western blot and immunofluorescence) according to the manufacturers' instructions, and validation data are available on manufacturers' websites.

Eukaryotic cell lines

Policy information about cell lines and Sex and Gender in Research

Cell line source(s)	Human embryonic kidney 293T cells (C6008) and human cervical cancer HeLa cells (C6330) were purchased from Beyotime. SRA01/04 cells were obtained from Dr. Jun Zhao's lab.
Authentication	The morphology of cells was checked at every passage for authentication purposes.
Mycoplasma contamination	Cells lines were confirmed to be mycoplasma-free.
Commonly misidentified lines (See ICLAC register)	No commonly misidentified lines were used in this study.

Animals and other research organisms

Policy information about studies involving animals; ARRIVE guidelines recommended for reporting animal research, and Sex and Gender in Research

Laboratory animals	Wistar rats aged 2 to 10 weeks were used in this study, depending on the specific experiment. Rats were housed under standard conditions (12 h light and 12 h dark cycles, 20–22 °C, 40–60% humidity) with food and water provided ad libitum.
Wild animals	No wild animals were used in this study.
Reporting on sex	Sex was not considered in the study design.
Field-collected samples	No samples were collected from the field.
Ethics oversight	Animal care and experimental protocols were approved by the Institutional Animal Care and Use Committee of the First Affiliated Hospital of Southern University of Science and Technology (protocol number 20200330-16) in accordance with the guidelines for the care and use of laboratory animals.

Note that full information on the approval of the study protocol must also be provided in the manuscript.

Plants

Seed stocks: n/a.

Novel plant genotypes: n/a.

Authentication: n/a.

Flow Cytometry

Plots

Confirm that:

☒ The axis labels state the marker and fluorochrome used (e.g. CD4-FITC).

☒ The axis scales are clearly visible. Include numbers along axes only for bottom left plot of group (a 'group' is an analysis of identical markers).

☒ All plots are contour plots with outliers or pseudocolor plots.

☒ A numerical value for number of cells or percentage (with statistics) is provided.

Methodology

Sample preparation: Samples were prepared as described in the "Methods" section.

Instrument: Agilent NovoCyte

Software: NovoExpress software

Cell population abundance: 10,000 events were collected and analyzed.

Gating strategy: FSC-A/SSC-A and FSC-A/FSC-H were applied to excluded debris and cell-cell complexes, respectively.

☒ Tick this box to confirm that a figure exemplifying the gating strategy is provided in the Supplementary Information.
